# Severe optic neuritis in a patient with combined neuromyelitis optica spectrum disease and primary Sjögren’s syndrome: a case report

**DOI:** 10.1186/1752-1947-6-401

**Published:** 2012-11-24

**Authors:** Petrina Tan, Wai Yung Yu, Thirugnanam Umapathi, Su-Ann Lim

**Affiliations:** 1 National Healthcare Group Eye Institute, Tan Tock Seng Hospital, 11 Jalan Tan Tock Seng, Singapore 308433, Singapore; 2 Department of Neuroradiology, National Neuroscience Institute, 11 Jalan Tan Tock Seng, Singapore 308433, Singapore; 3 Department of Neurology, National Neuroscience Institute, 11 Jalan Tan Tock Seng, Singapore 308433, Singapore

## Abstract

**Introduction:**

Optic neuritis, although uncommon, can be the initial presentation of Sjögren’s syndrome. Coexisting Sjögren’s syndrome has also been reported with neuromyelitis optica spectrum disorder. This case report highlights the association between the two diseases and the importance of rheumatological and neurological evaluations in patients with such diagnoses. Distinction of neuromyelitis optica with coexisting connective tissue disease has both prognostic and therapeutic significance for the patient.

**Case presentation:**

We report a case of a 56-year-old Chinese woman who presented with bilateral asymmetric visual loss secondary to optic neuritis. She was subsequently found to be seropositive for neuromyelitis optica immunoglobulin G (NMO-IgG) (anti-aquaporin-4 antibody) and was diagnosed with neuromyelitis optica spectrum disorder. She also fulfilled the international criteria for Sjögren’s syndrome. Despite initial high dose immunosuppressive therapy, she failed to regain vision in one eye.

**Conclusion:**

Patients presenting with optic neuritis and severe visual loss should be screened for neuromyelitis optica and treated appropriately. Neuromyelitis optica has been associated with systemic autoimmune diseases, in particular Sjögren’s syndrome, and current evidence indicates that they are two distinct entities. We recommend that both diagnoses be considered in cases of optic neuritis with severe visual loss.

## Introduction

Optic neuritis (ON), although uncommon, can be the initial presentation of Sjögren’s syndrome (SS) [1]. Coexisting SS has also been reported in 2% to 30% of patients with neuromyelitis optica (NMO) [2]. NMO is an inflammatory demyelinating disorder of the central nervous system (CNS) characterized by recurrent attacks of ON and longitudinally extensive transverse myelitis (LETM) distinct from multiple sclerosis. The discovery of neuromyelitis optica immunoglobulin G (NMO-IgG) (anti-aquaporin-4 antibody) and its specificity and sensitivity for NMO has led to its incorporation in the diagnostic criteria for NMO. In addition, incomplete forms of NMO (recurrent ON, LETM) and seropositivity for NMO-IgG predicts increased risk of progression to full spectrum NMO, resulting in the designations of these conditions as neuromyelitis optica spectrum disorder (NMOSD) [3]. In this article, we present a patient with ON who was found to have both primary SS and NMO-IgG seropositivity, suggestive of NMOSD and review the recent literature concerning the overlap between primary SS and NMOSD.

## Case presentation

**Figure 1:**
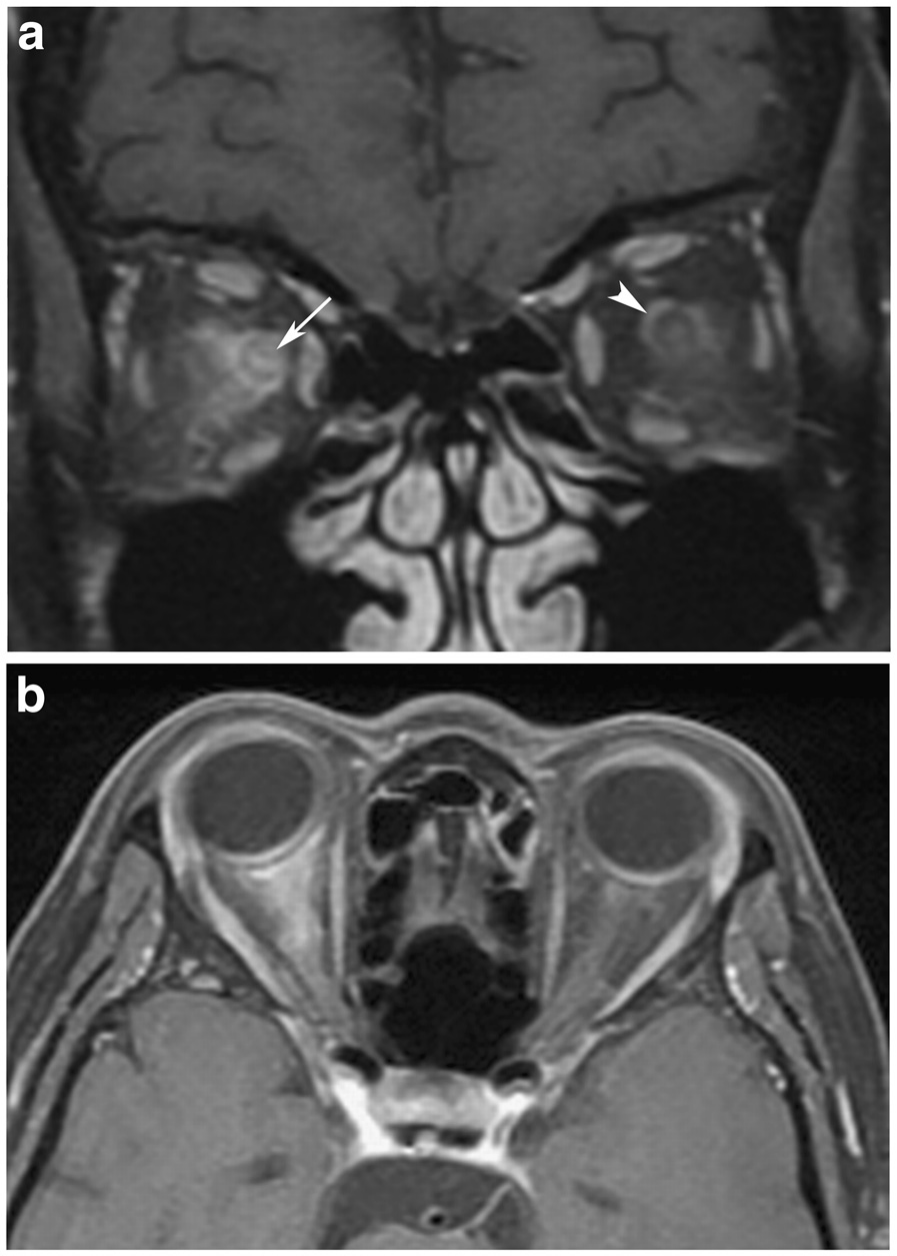
**Magnetic resonance imaging. (a)** Coronal post-gadolinium fat-saturated T1-weighted image shows enhancement and stranding in the intraconal fat bilaterally, worse on the right. The right optic nerve enhances (arrow head) along with enhancement of the optic sheath. Left perineural enhancement posterior to the globe is also present, as indicated by the arrow. **(b)**Axial post-gadolinium fat-saturated T1-weighted image demonstrated the marked enhancement in the right intraconal fat.

A 56-year-old Chinese woman with no previous history of ON, myelitis or comorbidities, complained of five days of right-sided headache and progressive visual loss initially only affecting the right eye but involving the left on the fifth day. The best-corrected visual acuity of her right eye at presentation was light perception and 6/7.5 on the left. There was right relative afferent pupillary defect with right disc swelling. The left disc was normal. Automated perimetry (24–2 threshold, Swedish Interactive Threshold Algorithm-standard strategy, Zeiss Carl Opthalmic Systems-Humphrey Division, Dublin, CA ) could not be done in the patient’s right eye. The test in her left eye was negative for any visual field defects (reliability indices-fixation losses: seven out of 16, false positive errors: 12%, false negative errors: 6%). A detailed ophthalmic examination was performed which included slit lamp examination of the anterior segment, dilated fundal examination, intraocular pressure measurement as well as fluorescein staining for corneal abnormalities. The ocular examination was negative for punctate epithelial erosions, uveitis, pars planitis or retinal vasculitis. There was no clinical evidence of transverse myelitis, and the patient declined spinal imaging due to financial constraints. Serological investigations revealed raised levels of the following autoimmune antibodies: anti-nuclear antibody, rheumatoid factor, anti-Ro antibody as well as NMO-IgG antibody. Neuroimaging showed swelling, edema and enhancement of the orbital segment of the right optic nerve with marked enhancement and stranding in the intraconal fat. There was also enhancement of the left optic sheath and intraconal fat posterior to the globe. No periventricular white matter plaques were found (Figure 1). A spinal tap showed normal levels of protein and inflammatory cells and oligoclonal bands were absent.

The patient had sicca symptoms for 12 years and the result of her Schirmer’s test was less than five mm in both eyes; thus fulfilling the international criteria for diagnosis of SS.

A diagnosis of NMOSD and primary SS was made. The patient was started on intravenous methylprednisolone (one mg/kg body weight/day) for five days. As there was no improvement in the vision of the patient’s right eye, plasmapheresis was initiated for the patient on the sixth day. Despite undergoing four cycles of plasma exchange, vision in the patient’s right eye was unchanged and the visual acuity of her left eye was 6/7.5. The right disc showed resolution of disc swelling after three months with subsequent temporal pallor.

## Discussion

This patient’s severe visual loss and degree of intraconal fat stranding was atypical for demyelinating ON or multiple sclerosis. She did not have any atherosclerotic risk factors or elevated levels of erythrocyte sedimentation rate to suggest non-arteritic and arteritic ischemic optic neuropathy respectively. The severe asymmetric bilateral optic neuropathy prompted a search for NMO and associated systemic autoimmune diseases such as SS.

CNS involvement in patients with Sjögren’s syndrome is much less common than peripheral nervous system involvement and ranges from 2% to 25% among patients [4,5]. Delalande *et al.* reviewed the neurologic manifestations in 82 patients with Sjögren’s syndrome and found that 13 out of 82 (16%) of patients had visual loss secondary to ON [1]. In another study by Gono *et al.*, three of 17 (18%) primary Sjögren’s syndrome patients had ON [5].

NMO is characterized by significant morbidity in more than 90% of cases and more than half of the affected patients will be unable to ambulate without assistance and/or be functionally blind by five years [6].

An association between NMO, NMOSD and systemic autoimmune disease has been reported in the literature. To date, 26 cases of overlap SS and NMO have been identified [6]. Present studies indicate that ON in patients with SS who are seropositive for NMO-IgG signifies the coexistence of two autoimmune diseases rather than a secondary vasculitic complication of the systemic disease. Pittock *et al.* studied NMO or NMOSD patients who may or may not have overlap SS or systemic lupus erythematosus (SLE) disease and compared them with a control group of SS and/or SLE patients [7]. NMO-IgG was detected only in patients with NMO or NMOSD with or without SS or SLE and was consistently negative in the controls. Jarius *et al.* also studied serum samples from 109 patients with established or possible systemic autoimmune disease, and vasculitis for the presence of NMO-IgG [8]. They found that NMO-IgG was present only in patients with neurological manifestations consistent with NMO, NMOSD, recurrent ON or LETM. Patients with other neurological manifestations were seronegative. Furthermore, NMO-IgG serum titres in patients with NMOSD and systemic autoimmune disease did not differ significantly from those in a group of unselected control patients with NMOSD but no systemic autoimmune disease. The above studies support the concept of NMO-IgG being involved in the pathogenesis of these neurological diseases and argue against it being part of the polyclonal B cell activation found in systemic autoimmune disease.

There is no current published literature regarding screening for SS in patients with NMO. However, Sánchez-Guerrero *et al.*[9] assessed the validity of several screening tests (European questionnaire for sicca symptoms [10], Schirmer’s test, and wafer test [11]) in a group of 336 ambulatory patients with chronic diseases. They found that the combined use of a model consisting of the European questionnaire with at least one affirmative answer, a positive result for Schirmer’s test of less than five mm in five minutes, and positive wafer test results had the best performance in terms of prediction for SS with a likelihood ratio of 9:4. Given the suitability, ease of administration, low cost and minimal discomfort of the tests, they recommended their use in parallel to identify SS in ambulatory patients with chronic diseases. The availability and use of these tests may vary among different institutional practices; however, it is useful to keep in mind these tests when faced with NMO patients with possible SS.

There are therapeutic implications of the diagnosis of NMO and the interrelationships between NMO and/or NMOSD and systemic autoimmune disease. Recognition of the distinct entity of NMO and/or NMOSD from multiple sclerosis (MS) is important because treatment recommended for MS-like ON is not applicable. Patients with NMO-IgG seropositivity have been known to have a high risk of relapse at 50% or greater, and may need maintenance therapy with azathioprine for a minimum of five years [12]. Our patient was advised on long-term immunosuppression with azathioprine; however, she declined treatment due to financial issues. She has remained relapse-free for the past 22 months. It is possible that control of autoimmune disease may be beneficial for the treatment of NMOSD. Although treatment strategies for NMO and/or NMOSD and systemic autoimmune disease are similar and may overlap, care must be taken when treatment involves use of biologic agents targeting tumor necrosis factor and its receptors because there have been reports of associated CNS demyelinating events. Their specific effects on NMO and/or NMOSD are still unknown.

## Conclusion

Current literature suggests NMO as a distinct entity from MS requiring different treatment modalities; as such, patients presenting with ON and severe visual loss should be screened for NMO and treated appropriately. Less commonly, ON may be the initial presentation of SS. Rheumatologic evaluation should be undertaken in patients with evidence of clinical and serological autoimmunity. NMO has been associated with systemic autoimmune diseases, in particular SS, and current evidence indicates that they are two distinct entities. Further studies are required to better understand the impact of these systemic diseases and their treatment on the morbidity of NMO and NMOSD.

## Consent

Written informed consent was obtained from the patient for publication of this case report and accompanying images. A copy of the written consent is available for review by the Editor-in-Chief of this journal.

## Competing interests

The authors declare that they have no competing interests*.*

## Authors’ contributions

PT was involved in the care of the patient, and was a major contributor in writing the manuscript and literature review. WYY contributed to the figures in the manuscript. TU was involved in the care of the patient and editing the manuscript. SAL was involved in the care of the patient and editing of the manuscript. All authors read and approved the final manuscript.
